# Assessing the application of American Heart Association (AHA) guidelines in the management of heart failure with reduced ejection fraction

**DOI:** 10.1186/s43044-025-00629-z

**Published:** 2025-03-10

**Authors:** Sima Sobhani Shahri, Zahra Pirayesh, Azar Zare Noughabi, Marzieh Heshmati, Saeede Khosravi Bizhaem, Shima Jafari, Toba Kazemi

**Affiliations:** 1 Student Research Committee, Birjand University of Medical Sciences, birjand, Iran; 2 Cardiovascular Diseases Research Center, Birjand University of Medical Sciences, Birjand, Iran

**Keywords:** Heart failure with reduced ejection fraction (HFrEF), Guideline treatment adherence, Sodium–glucose cotransporter-2 inhibitors (SGLT2I), Heart failure treatment, American Heart Association (AHA)

## Abstract

**Background:**

Heart failure (HF) is a significant global health issue. Appropriate and timely treatment at target doses significantly reduces mortality and enhances quality of life. However, studies indicate suboptimal pharmacotherapy among patients. This study aims to assess the medical treatment of patients with heart failure and reduced ejection fraction (HFrEF) and their adherence to the American Heart Association (AHA) guidelines. The study was designed as a cross-sectional analysis in the cardiac department of Razi Hospital in Birjand from March 20, 2020, to March 11, 2023, focusing on patients with left ventricular ejection fraction less than or equal to 40%. Data were extracted from patients’ medical records. Medications were classified according to the four-pillar therapy recommended by the AHA, including *β*-blockers, ARNI, ACE inhibitors/ARBs, SGLT2, and MRAs. Patients were grouped based on their treatment regimens. The percentage of achieved target doses for each medication was categorized as follows: 0–25%, 25–50%, 50–99%, and 100%. Statistical analysis was conducted using SPSS version 22.

**Results:**

The study included patients with a mean age of 66 ± 13.7 years, of whom 278 (69%) were male. The mean ejection fraction was 26.8 ± 9.6%, and the most prevalent comorbidity was coronary artery disease (CAD) observed in 68.0% of patients. The in-hospital mortality rate was 5%. The results revealed that only 20% were on quadruple therapy, while 10% received none of the recommended medications. The prescription rates for key medications were as follows: *β*-blockers 76.4%, ACE inhibitors/ARBs 71.6%, MRA 63.3%, SGLT2I 33.5%, and ARNI 0%. Notably, 94.8% of prescribed SGLT2I doses met the target dose, while 84.4% of *β*-blocker prescriptions and 61.8% of ACEI/ARB prescriptions were below 75% of the target dose.

**Conclusion:**

The findings reveal significant gaps in the prescription of essential therapies, including MRAs and ARNIs, which are crucial for managing myocardial dysfunction. Addressing these gaps underscores the necessity for ongoing education and training for healthcare providers in heart failure management.

## Background

Heart failure (HF) is characterized by insufficient cardiac output to meet the body’s needs and/or increased intraventricular filling pressure. This condition manifests through symptoms such as dyspnea, orthopnea, paroxysmal nocturnal dyspnea, fatigue, and ankle swelling. An estimated 6.5 million U.S. adults are affected by heart failure. Approximately 1 million hospitalizations occur annually due to heart failure, with about 50% attributed to HFrEF [1]. The burden of heart failure, especially in low- and middle-income countries, Eastern Mediterranean region (EMR) like Iran, is substantial, with rising prevalence and high mortality rates linked to various modifiable predictors [2, 3]. In Iran, the 1-year mortality rate for heart failure is reported at 32%, which aligns with mortality rates observed in other countries [2].

Current treatment guidelines for heart failure with reduced ejection fraction (HFrEF) include over ten different guidelines-directed medical therapies (GDMT), notably those from the American College of Cardiology/American Heart Association (AHA) and the European Society of Cardiology [4, 5]. These guidelines universally recommend the use of beta-blockers, RAAS blockers (angiotensin-converting enzyme (ACE) inhibitors, and angiotensin II receptor blockers (ARBs)), mineralocorticoid receptor antagonists (MRAs), and sodium–glucose cotransporter-2 (SGLT-2) inhibitors as the cornerstone of HFrEF treatment [4–6]. Early initiation of these medications at target doses has been shown to reduce mortality, prevent rehospitalization, and improve the quality of life for patients with reduce ejection fraction [6].

However, evidence indicates that guideline recommendations are often not adequately implemented, resulting in suboptimal pharmacotherapy for HF. Some patients do not receive any guideline-directed medical therapy (GDMT) medications, and prescribed dosage frequently fall below 50% of the target dose [7–9]. Factors contributing to this issue include the risk of side effects, treatment discontinuation, economic constraints, insurance challenges, and prolonged treatment duration [8]. While establishing heart failure clinics could help mitigate the burden of this condition, there are significant challenges to implementing such facilities, particularly in underprivileged areas. The lack of resources, infrastructure, and healthcare personnel in these deprived regions hinders the development of specialized care centers, which are essential for effective management and treatment of heart failure [9].

Given the limited research on HFrEF treatment adherence to GDMT, particularly in Iran, this study was designed to assess the adherence to medical treatment guidelines among hospitalized patients with HF at the time of discharge from the cardiac department.

## Methods

### Study design and population

The present study was designed as a retrospective hospital-based study, including patients with heart failure with reduced ejection fraction (HFrEF) admitted to the cardiac department of Razi Hospital in Birjand from March 20, 2020, to March 11, 2023. Following approval from the Research Ethics Committee (IR.BUMS.REC.1402.185), patients were selected through convenient sampling. This approach involved selecting patients based on their availability and willingness to participate during the study period. To identify eligible patients, we utilized the ICD-10 code I50.x for heart failure diagnosis. According to the American College of Cardiology, American Heart Association, and Heart Failure Society of American guidelines (2022), HFrEF is defined as an ejection fraction (EF) of ≤ 40% [4].

### Data collection

We referred to the archives and extracted the following information from the patients' records: the assistant's history sheet, types of medications and their doses from the physician's orders, laboratory test results, and echocardiography reports were available in the records. The following variables were recorded: age, gender, EF level, underlying diseases, functional class according to the New York Heart Association (NYHA), length of hospitalization, mortality rate, and the names and dosages of medications recommended in the guidelines-directed medical therapy (GDMT) as well as other cardiac-related medications.

### Guideline-directed medical therapy

Following the AHA’s four-pillar therapy model, which includes beta-blockers, ARNI (angiotensin receptor–neprilysin inhibitor), ACE inhibitors/ARBs, SGLT2 inhibitors, and MRAs, patients were categorized based on their treatment regimens. Patients were classified into five treatment categories: no therapy, single therapy (individual agents such as beta-blockers, ARNI, ACE inhibitors/ARBs, SGLT2 inhibitors, or MRAs), double drug therapy, triple drug therapy, and quadruple therapy (combination of ACE inhibitors/ARBs, beta- blockers, MRAs, and SGLT2 inhibitors).

Additionally, the dosages of specific medications—SGLT2 inhibitors (empagliflozin), ACE inhibitors (captopril and enalapril), ARBs (losartan and valsartan), beta-blockers (carvedilol, bisoprolol, and metoprolol), and MRAs (spironolactone and eplerenone)—were analyzed. The percentage of target doses achieved was categorized into four ranges: 0–25% of the target dose, 25–50%, 50–99%, and 100% of the target dose, as recommended by the American College of Cardiology, American Heart Association, and Heart Failure Society of American guidelines (2022) [4].

### Data analysis

In this study, we conducted a descriptive analysis. Categorical variables are presented as frequencies and percentages, while continuous variables are presented as mean ± standard deviation (SD). All data analysis was performed using SPSS version 22.

## Results

This study included 403 patients diagnosed with heart failure with reduced ejection fraction (HFrEF).

The mean age of the patients was 66 ± 13.7 years, with 278 (69%) being male. Over half of the patients (52.1%) were classified in NYHA classes II or III, and the mean ejection fraction (EF) was 26.8 ± 9.6%. The in-hospital mortality rate was 5%, and the duration of hospitalization was 4.3 ± 3.2 days.

The frequency of comorbidities among the patients included: coronary artery disease (CAD), 68.0%; hypertension, 49.9%; diabetes mellitus (DM), 26.8%; and anemia 23.8%. Notably, 56.6% of the patients had at least two comorbidities, while 9.6% reported no underlying diseases.

The most frequently abnormal laboratory parameter was high fasting blood sugar (FBS), observed in 28.6% of the patients (Table 1).

**Table 1:** Baseline characteristics and laboratory examinations

Variables	Total (*n* = 403)
Age (years)	66.0 ± 13.7
Gender (male)	278 (69.0)
Smoking	44 (10.9)
Addiction	104 (25.8)
Comorbidities	
Coronary artery disease	274 (68.0)
Hypertension	201 (49.9)
Diabetes mellitus	108 (26.8)
Dyslipidemia	102 (25.3)
Anemia	96 (23.8)
Atrial fibrillation (AF)	19 (4.7)
Chronic kidney disease	7 (1.7)
Chronic obstructive pulmonary disease (COPD)	41 (10.2)
Ejection fraction (EF)	26.8 ± 9.6 (7–40%)
EF category	
40–30%	196 (48.6)
30–15%	160 (39.7)
< 15%	47 (11.7)
NYHA functional class	
Class I	82 (20.3)
Class II	114 (28.3)
Class III	96 (23.8)
Class IV	55 (13.6)
Duration of hospitalization (days)	4.3 ± 3.2
Mortality rate	22 (5.5)
Laboratory examination	
Sodium (Na, meq/L)	135.9 ± 72.1
Sodium < 135	72 (17.9)
Potassium (K, meq/L)	4.3 ± 0.6
Potassium < 3.5	15 (3.7)
Potassium > 5.2	20 (5.0)
Fasting blood sugar (FBS, mg/dL)	135.9 ± 72.1
FBS ≥ 126	115 (28.6) (126–562)
Urea (mg/dL)	52.5 ± 33.4
Creatinine (Cr, mg/dL)	1.2 ± 0.9
Cr < 1.1 in female and < 1.2 in male	118 (29.3)
Mean corpuscular volume (MCV, fL)	86.4 ± 9.3
MCV < 80	31 (7.7)

Data presented as frequency (%) and mean ± SD

As detailed in Table 2, the rates of guideline-recommended medications utilized in HFrEF treatment were as follows: beta-blockers: 76.4%; ACE inhibitors/ARBs: 71.6%; MRAs: 63.3%; SGLT2Is: 33.5%; and an ARNI: 0%. It is noteworthy that two patients received both an ACE inhibitor and an ARB, and three patients were treated with two different ARBs (losartan and valsartan). Additionally, three patients received two different types of beta-blockers (carvedilol and bisoprolol), while atenolol, which is contraindicated in heart failure, was prescribed for one patient.

**Table 2:** Type and doses of guideline-recommended medications for the treatment of heart failure

Medicine	Frequency (%)	Dosage mean (mg/day)
ARNI	0	0
ACE inhibitors	57 (14.1)	
Enalapril	25 (6.2)	6.4 ± 5.7
Captopril	31 (7.7)	25.1 ± 14.4
Lisinopril	1 (0.2)	5.0
ARBs	234 (58.1)	
Valsartan	37 (6.2)	123.7 ± 56.0
Losartan	200 (49.6)	32.5 ± 18.9
Beta-blockers	308 (76.4)	
Carvedilol	185 (45.9)	9.6 ± 4.5
Bisoprolol	114 (28.3)	4.2 ± 3.0
Atenolol	1 (0.2)	50.0
Metoprolol	13 (3.2)	42.8 ± 24.4
Mineralocorticoid receptor antagonists (MRAs)	255 (63.3)	
Eplerenone	9 (2.2)	26.3 ± 9.7
Spironolactone	248 (61.5)	24.7 ± 7.1
SGLT2 inhibitors (Empagliflozin)	135 (33.5)	
Empagliflozin with metformin	50 (12.4)	11.9 ± 3.7
Empagliflozin	86 (21.3)	9.9 ± 0.5

Data presented as frequency (%) and mean ± SD

Among other cardiac medications listed in Table 3, the most frequently prescript were: atorvastatin: 72.0%; aspirin: 73.4%; anticoagulants: 64.4; and loop diuretics: 44.4%. Most of these medications were prescribed at target doses.

**Table 3:** Other prescribed medications for heart failure patients

Medicine	Frequency (%)	Dosage mean (mg/day)
*Loop diuretic*		
Furosemide	179 (44.4)	42.4 ± 22.6
*Anti-arrhythmic*	52 (12.9)	
Digoxin	29 (7.2)	0.33 ± 0.3
Amiodarone	23 (5.7)	167.4 ± 73.2
*Anticoagulant*	50 (12.5)	
Rivaroxaban	26 (6.5)	12.3 ± 4.7
Apixaban	24 (6.0)	5.8 ± 2.9
*Antiplatelet*	309 (76.6)	
Aspirin	296 (73.4)	80
Clopidogrel	209 (51.9)	75
*Other*		
Atorvastatin	290 (72.0)	39.5 ± 14.4
Amlodipine	23 (5.7)	8.1 ± 4.5
Hydralazine	5 (1.2)	32.5 ± 16.7
Diltiazem	2 (0.5)	45.0 ± 21.2
Hydrochlorothiazide	2 (0.5)	25
Nitroglycerin	215 (53.3)	4.5 ± 1.9

Data presented as frequency (%) and mean ± SD

**Fig. 1:**
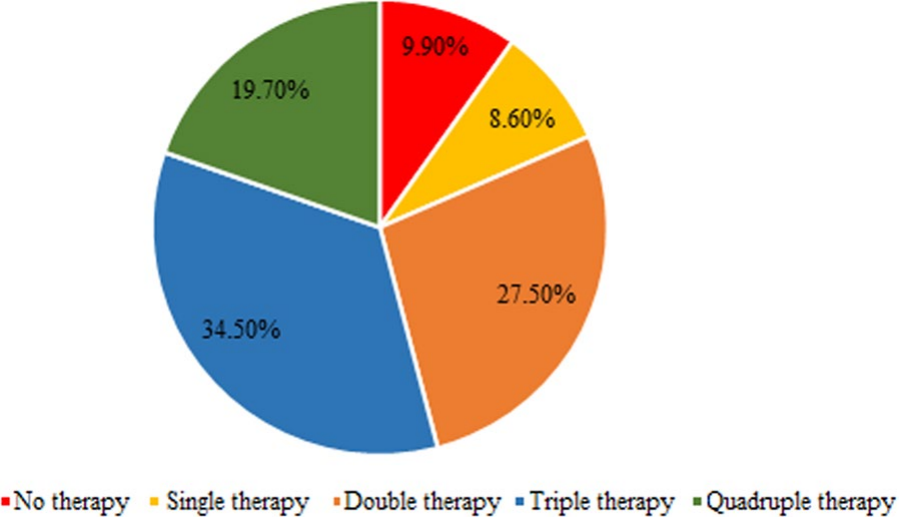
Treatment pattern and pharmacotherapy achieved by patients with HErEF

Regarding treatment pattern and combination therapies, as shown in Fig. 1, 20% of the patients were on quadruple therapy, while 10% did not receive any of the recommended medications. Triple therapy was the most commonly prescribed regimen, with ACE inhibitors/ARBs and MRAs being the most frequently reported combinations (Fig. 1).

**Fig. 2:**
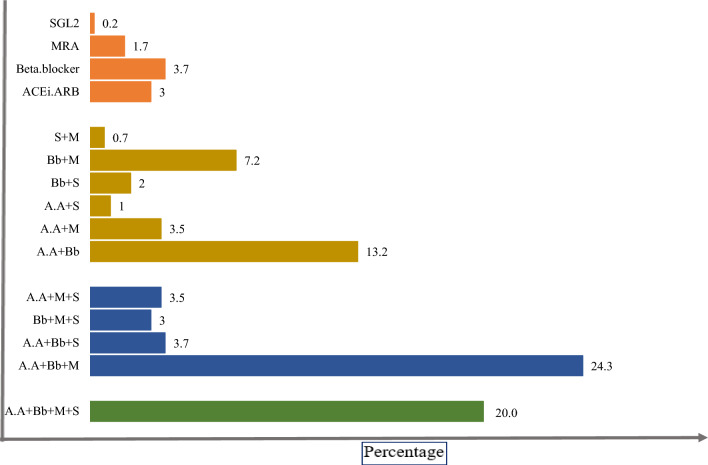
Summary of medicine prescription to compare with guideline. Abbreviations: S, SGL2I; M, mineralocorticoid receptor antagonists (MRA); Bb, beta-blockers; and A.A, angiotensin-converting enzyme (ACE i) inhibitors. Angiotensin II receptor blockers (ARB)

SGLT2I was the most commonly prescribed medication within guideline recommendations. However, 84.4% of prescribed beta-blockers and 61.8% of ACE inhibitors/ARBs were administered at less than 25% of the target dose (Table 4 and Fig. 2) [4].

**Table 4:** Comparison of the doses of prescribed medications to the target dose

Medicine group	0–25%	25–50%	50%–99%	100%
ACEI/ARB	178 (61.8)	99 (34.2)	7 (2.4)	4 (1.4)
MRA	25 (9.8)	221 (86.7)	–	9 (3.5)
Beta-blocker	260 (84.4)	34 (11.0)	–	14 (4.5)
SGLT2I	–	–	7 (5.1)	128 (94.8)

Data presented as frequency (%)

**Fig. 3:**
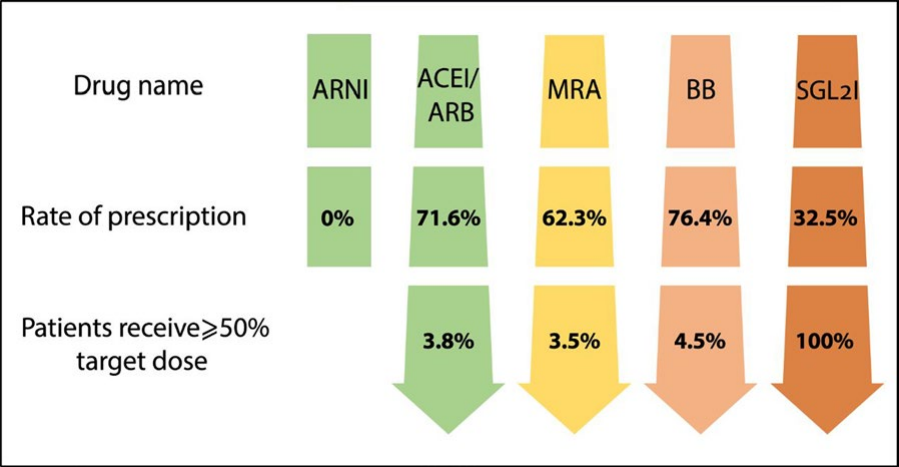
Prescription rates of medications according to the four pillars of the AHA guidelines for heart failure management

Figure 3 illustrates the prescription rates of medications according to the four pillars of the American Heart Association (AHA) guidelines for heart failure management. As shown in Fig. 3, none of our patients were prescribed any medications from the ARNI group [4].

## Discussion

The present study was a retrospective study involving 403 patients with HFrEF who were admitted to the cardiac department. The mean age of them was 66 ± 13.7 years, which is younger compared to many studies conducted in Sweden, the Netherlands, and the USA [10–12]. The prevalence of CAD was noted to be the most common comorbidity at 68%, with an anticoagulant prescription rate of 12.5% and antiplatelet therapy at 76.6%. These findings underscore that ischemic disease is the predominant etiology of HF in our patient population, consistent with other studies [13, 14].

More than half of the participants in our study exhibited moderate symptoms, categorized as NYHA functional class II and III. The in- hospital mortality rate was 5%, aligning closely with findings from Bollmann’s study in Germany (5.5%), Dai’s study in Canada (4.9%), and the intermediate-risk patients in Kwok’s study in the USA (5.94%) [15–17]. In contrast, Park’s reported a lower mortality rate of 2.2% [14]. Generally, the presence of comorbidities, renal dysfunction, electrolyte disturbance, advanced age, and decreased cardiac function can all contribute to an increased risk of mortality [16].

From the guideline-recommended medications, beta-blockers had the highest prescription rate at 76.4% in our study. Comparable studies report similar rates ranging from 67 to 87% [14, 19, 20]. Beta-blockers provide anti-arrhythmic and anti-ischemic effects and inhibit renin release, ultimately reducing cardiovascular mortality and preventing rehospitalization [21, 22]. Notably, 23.6% of patients in our study did not receive beta-blockers, which may be attributed to comorbidities such as asthma or COPD as well as potential side effects such as hypotension and bradycardia [23].

Renin–angiotensin–aldosterone system (RAAS) blockers, including ACE inhibitors (ACEIs) and angiotensin II receptor blockers (ARBs), are also recommended medications that reverse cardiac remodeling, decrease systemic vascular resistance, and improve vascular compliance, all of which are associated with lower mortality rate [24, 25]. In our study, the prescription rate for ACEIs/ARBs was 72.2%, which aligns with the previous findings [14, 18, 19, 26] but differs from Brunner-La study (84%) [11] and Chakrala’s study (81.7%) [20]. Among our patients, 58.1% received ARBs while only 14.1% were treated with ACEIs. The latter may be associated with poor tolerance of ACEIs, as common adverse effects include cough, hypotension, and worsening renal function [26].

In our study, MRAs were prescribed to 63.3% of patients, which aligns with other studies reporting prescription rates ranging from 60 to 88% [19, 27]. MRAs are known to mitigate inflammation and fibrosis within cardiac tissue, thereby improving myocardial dysfunction [28]. However, the observed abnormalities in creatinine levels (29.3%), renal dysfunction (1.7%), or hyperkalemia (5%) among our patients may contribute to the absence of MRA prescriptions in the remaining 36.7% of cases.

In the present study, the prescription rate of SGL2I was 33.5%. Other studies have reported varied rates depending on the treatment center, ranging from 0 to 100%. For instance, prescription rates were 25% in the USA [29] and 13.7% in South Korea [30]. Empagliflozin is the only SGL2I available in Birjand and can be prescribed either independently or in combination with metformin (Synoripa). Despite a significant global increase in SGLT2I prescriptions over the past 6 years [31], this class of medication remains one of the most neglected guideline- recommended treatments in our setting. This could be partly attributed to high medication costs and limited insurance coverage in Iran.

Angiotensin receptor–neprilysin inhibitors (ARNI) have demonstrated significant benefits in reducing cardiovascular mortality, rehospitalization, and overall morbidity in patients with chronic heart failure [32]. However, despite these advantages, our study reported a prescription rate of 0% for ARNI. This lack of prescription may be attributed to several factors, including insufficient awareness among healthcare providers, potential barriers in access to the medication, and concerns regarding cost and reimbursement.

In the present study, approximately 20% of the overall patients were discharged with prescriptions for quadruple medical therapy, which included SGLT2 inhibitors, ACE inhibitors/ARBs, beta-blockers, and mineralocorticoid receptor antagonists (MRAs). This combination of medications has been shown to reduce mortality rates, decrease hospital admission for heart failure, and extend survival [33]. The previous studies have indicated that 80.0% of patients do not receive all four cornerstone classes of drugs [23, 34]. However, it is concerning that 10% of patients received none of the recommended therapies.

The rate of triple therapy was 24.7%, which is consistent with earlier studies [11, 19, 35] but lower than more recent study, such as Degrade et al. study [36] at 46.5%.

In our findings, the majority of prescribed beta-blockers (84.4%) and RAAS blockers (61.4%) were dosed at ≤ 25% of the target dose, while approximately 86.7% of prescribed MRAs were dosed between 25 and 50% of the target. In contrast, 94.8% of prescribed SGLT2 inhibitors were at 100% of the target dose. The observed gap between actual prescriptions and target doses can be attributed to several factors: patient-related characteristics, including medical and socio-demographic factors such as sex, age, severity of heart failure, and socioeconomic status; treatment-related aspects, such as tolerability and medication side effects; and healthcare-related factors that influence the delivery and quality of care for patients with heart failure [9, 19, 37–39].

## Limitations

This study has several limitations. The retrospective design depends on the accuracy and completeness of available medical records, which may introduce biases. Additionally, confounding variables, such as socioeconomic status and medication adherence, were not controlled for, potentially impacting the outcomes.

## Conclusions

The findings from this study highlight significant gaps in the prescription of essential therapies for patients with heart failure and reduced ejection fraction (HFrEF), particularly concerning mineralocorticoid receptor antagonists (MRAs) and angiotensin receptor–neprilysin inhibitors (ARNI). While adherence to the American Heart Association (AHA) guidelines is critical for optimizing treatment outcomes, it is essential to recognize that these guidelines are designed to serve as valuable tools to inform treatment plans rather than compulsory requirements for every patient.

Clinical judgment should remain at the forefront of decision-making, allowing healthcare providers to tailor treatments based on individual patient circumstances. However, our analysis indicates that many patients remain on suboptimal pharmacotherapy regimens, which may adversely affect their morbidity and mortality rates. The observed discrepancies underscore the need for ongoing medical education for healthcare providers regarding current guidelines and the importance of adhering to evidence-based practices in heart failure management.

Ultimately, improving adherence to guideline-recommended therapies is crucial for enhancing the quality of care and outcomes for patients with HFrEF. Future interventions should focus on bridging the gap between guidelines and clinical practice to ensure that all patients receive the most effective treatments.

## Data Availability

No datasets were generated or analyzed during the current study.

## Abbreviations

HF: Heart failure
AHA: American Heart Association
HFrEF: Heart failure with reduced ejection fraction
GDMT: Guidelines-directed medical therapies
NYHA: New York Heart Association
RAAS blockers: Renin–angiotensin–aldosterone system blocker
ACE i: Angiotensin-converting enzyme inhibitors
ARBs: Angiotensin II receptor blockers
MRAs: Mineralocorticoid receptor antagonists
SGLT-2: Sodium–glucose cotransporter-2
ARNI: Angiotensin receptor–neprilysin inhibitor
CAD: Coronary artery disease
EF: Ejection fraction
